# Prostate Cancer and Bone Metastases: The Underlying Mechanisms

**DOI:** 10.3390/ijms20102587

**Published:** 2019-05-27

**Authors:** Sok Kuan Wong, Nur-Vaizura Mohamad, Tijjani Rabiu Giaze, Kok-Yong Chin, Norazlina Mohamed, Soelaiman Ima-Nirwana

**Affiliations:** Department of Pharmacology, Faculty of Medicine, Universiti Kebangsaan Malaysia, Jalan Yaacob Latif, Bandar Tun Razak, Cheras, Kuala Lumpur 56000, Malaysia; jocylnwsk@gmail.com (S.K.W.); vaizuramohd@gmail.com (N.-V.M.); rtgiaze1143@gmail.com (T.R.G.); chinkokyong@ppukm.ukm.edu.my (K.-Y.C.); azlina@ppukm.ukm.edu.my (N.M.)

**Keywords:** hormone, inflammation, osteoprotegerin, estrogen

## Abstract

Patients with advanced prostate cancer often develop bone metastases, leading to bone pain, skeletal fracture, and increased mortality. Bone provides a hospitable microenvironment to tumor cells. The disease manifestation is driven by the interaction between invading tumor cells, bone-forming osteoblasts, and bone-resorbing osteoclasts. The increased level of osteoclast-activating factor (parathyroid hormone-related peptide, PTHrP) is believed to induce bone resorption by upregulating receptor activator of nuclear factor-kappa B ligand (RANKL) and the release of various growth factors into the bone microenvironment to enhance cancer cell growth. However, the underlying molecular mechanisms remain poorly understood. This review outlines the possible molecular mechanisms involved in governing bone metastases driven by prostate cancer, which further provide the basis in searching for new molecular targets for the development of potential therapy.

## 1. Introduction

Prostate cancer is cancer that occurs in the prostate gland, which is located below the bladder and in front of the rectum. It is the most common cancer affecting men. Approximately 26.5% of the male population has been diagnosed with prostate cancer worldwide, and about 13.7% were diagnosed within the Asia-Pacific region in the year 2008 [1]. Although the risk of developing prostate cancer increases dramatically after the age of 50, most cases of prostate cancer are diagnosed after the age of 65 [1].

In the earlier stage of advanced prostate cancer, malignant cells shed from the primary tumor migrate locally, invade blood vessels, and may disperse widely in the body. Prostate cancer cells that spread out of the prostate show an exquisite tropism for the bone. In one autopsy study, approximately 90.1% of the men who had died with hematogenous metastases of prostate cancer were diagnosed with bone metastases [2]. Based on the “seed and soil” theory proposed by Paget, the growth of tumor foci tends to be the direct result of a specific organ’s microenvironment [3]. Prostate cells (“seeds”) in the bloodstream need to settle in an appropriate “soil”, thus they preferentially migrate to bone (as a hostland) [4,5,6]. These malignant cells invade and eventually proliferate in the bones of the axial skeleton, such as the ribs, pelvis, and spine, where red marrow is most abundant [4].

During metastatic bone disease, the interaction between tumor cells with osteoblasts and osteoclasts elicits an osteolytic, osteoblastic, or mixed bone response [7]. A purely osteolytic response is characterized by the destruction of normal bone attributable to the occurrence of osteoblast inactivation as well as osteoclast recruitment and activation in the tumor-bone microenvironment. Osteolytic lesions are characterized by soft sections of damaged bone resulting from an osteolytic response that can cause bone pain and fractures. A purely osteoblastic response refers to the deposition of new bone due to the new bone formation which is not preceded by bone resorption [8]. Osteoblastic lesions, resulting from an osteoblastic response, are depositions of mineralized or calcified bone into the tissue lesions. A mixed bone response is a condition whereby an individual experiences a combination of both osteolytic and osteoblastic components.

Bone is a unique and conducive microenvironment that provides a milieu for metastatic cancer cells to colonize and thrive. The “vicious cycle” hypothesis has been developed to explain the process of cancer cell metastases to bone [9]. The production of parathyroid hormone-related peptide (PTHrP) by tumor cells up-regulated receptor activator of nuclear factor-kappa B ligand (RANKL), and down-regulated osteoprotegerin (OPG) by osteoblast to activate osteoclastogenesis and bone resorption. Accelerated bone resorption, in turn, promotes the release of bone-derived growth factors such as transforming growth factor-beta (TGF-β), the insulin-like growth factor (IGF)-1, and raised extracellular calcium concentration to further support the growth of cancer cells [9,10]. However, the mechanism of action of bone metastases development from prostate cancer remains to be elucidated, but there is increasing evidence from preclinical and cell culture studies.

In this review, we aim to describe the signaling molecules critical for the progression of prostate cancer into the bone microenvironment. A better understanding of the pathophysiology of prostate cancer bone metastases serves as a basis in searching for potential therapeutic management. We believe that targeting these signaling molecules may be potential strategies in the prevention or treatment of prostate cancer bone metastases.

## 2. The Underlying Molecular Mechanisms in Prostate Cancer Bone Metastases

Various types of prostate cancer cell lines, including LuCaP 23.1, LNCaP, C4-2, and IGR-CaP1, were utilized as prostate cancer models. The LuCaP 23.1 and LNCaP cells are highly sensitive to androgen [11,12]. The C4-2 cell lines showed features of reduced androgen sensitivity and increased metastatic capability [13]. In the androgen-sensitive (androgen-dependent) prostate cancer cell lines, the downregulation in androgen receptor (AR) expression reduced AR-mediated transcription and cell growth. Meanwhile, the knockdown of AR expression had a marked effect on AR-mediated transcription and cell growth in the androgen-insensitive (androgen-independent) prostate cancer cell lines [14]. The expression of AR is an important regulator of prostate cancer cell growth and development at the early stage. However, prostate cancer progresses to castration-resistant prostate cancer at the later stage. Thus, possible correlation between AR expression and the signaling molecules involved in prostate cancer bone metastasis could be considered. On the other hand, the IGR-CaP1 cell line represents a unique model recapitulating widespread bone metastasis with mixed osteoblastic and osteolytic bone lesions that resemble the conditions observed in patients [15].

### 2.1. The Role of Parathyroid Hormone (PTH)

Parathyroid hormone (PTH) is a hormone secreted by the parathyroid gland which plays an important role in bone remodeling. It stimulates bone resorption by osteoclasts indirectly through PTH binding receptors located on osteoblasts. Upon binding of PTH on osteoblasts, the expression of OPG is downregulated whereas the expression of RANKL is upregulated [16]. Signaling to the bone marrow-derived osteoclast precursors, high levels of RANKL consequently stimulate their fusion, differentiation, and activation. PTH causes a net bone loss through an increased resorption process when administered in a continuous fashion, but a net bone gain through an enhanced formation process when administered intermittently. To our knowledge, only a handful of evidence documented the ectopic expression of PTH by the thyroid [17,18] and other non-parathyroid tumors [19,20,21]. Specifically, studies on the ectopic expression of PTH by prostate tumors are limited [22].

Another member of the parathyroid hormone family, PTHrP, shares a common ancestry and high amino-acid sequence similarity in the N-terminal region with other members of the group that enables it to bind and activate the PTH receptor directly in order to stimulate osteoclast and osteoblast activity [23,24,25]. Thus, PTHrP has been suggested to have a crucial role in skeletal metastasis of prostate carcinoma. A study by Blomme et al. investigated the effects of PTHrP overexpression on tumor growth and the incidence of bone metastases in rats induced with MatLyLu prostate adenocarcinoma cells (containing a full-length rat PTHrP cDNA). The results showed that all rats injected with 20,000 MatLyLu cells successfully developed osteolytic metastases in the long bones and vertebrae after 16 days. However, PTHrP failed to induce any significant differences in the size of metastasis foci or tumor cell proliferation [26]. A similar study by Rabbani et al., using a syngeneic rat of MatLyLu prostate cancer cells with intracardiac inoculated PTHrP, led to lumbar vertebral metastasis and consequent hind-limb paralysis. This study found an increase in osteoclastic activity with PTHrP observed from a histological examination [27]. These findings proposed that tumor-derived PTHrP played a critical role in skeletal metastasis by forming a vicious cycle through enhancement of the bone remodeling pathways. Liao et al. then showed that PTHrP overexpression induced higher growth rates in the ACE-1 canine prostate cancer cell line and generated larger tumors when inoculated subcutaneously (5 × 10^3^ prostate cancer cells) in athymic mice. Histology results revealed increased bone mass adjacent to PTHrP overexpressing tumor foci, with increased osteoblastogenesis (evidenced by alkaline phosphatase (ALP) staining) and osteoclastogenesis (evidenced by tartrate-resistant acid phosphatase (TRAP) staining) [28]. Overall, these findings collectively indicated that PTHrP is an osteolytic and osteoblastic factor which is highly expressed in bone metastases of prostate cancer.

### 2.2. The Role of the RANK/RANKL/OPG System

The receptor activator of nuclear factor-kappa B (RANK)/RANKL/OPG system is a key molecular system discovered to regulate the bone modeling and remodeling process. Osteoprotegerin is a decoy receptor produced by osteoblasts that blocks the association between RANKL and RANK, thus inhibiting osteoclastogenesis and increasing bone mass. Apart from controlling the normal bone metabolism, this system also plays an essential role in pathological bone metabolism, such as metastatic disease in bone.

Some studies suggested a potential role of OPG in the treatment of prostate cancer with bone metastases. A study by Corey et al. demonstrated that four- to six-week-old severe combined immunodeficient (SCID) mice, intratibially injected with OPG-transfected C4-2 cells (a subline of prostate cancer cells), had higher bone mineral density (BMD), percentage of trabecular bone volume, as well as decreased osteoblast and osteoclast numbers compared to the animals intratibially injected with pcDNA-C4-2 tumors. The data implied that the expression of OPG inhibited bone lysis is associated with C4-2 bone metastases, resulting in a net increase in bone volume [29]. In addition, Kiefer et al. utilized six-week-old Fox Chase SCID male mice to investigate the effects of OPG on osteoblastic prostate cancer metastases. The animals were intratibially injected with prostate cancer (LuCaP 23.1) cells and subcutaneously administered with osteoprotegerin human recombinant protein (Fc-OPG) (6.0 mg/kg) thrice a week, either 24 h pre-injection or four weeks post-injection. The findings indicated that Fc-OPG did not inhibit osteoblastic bone lesions of LuCaP 23.1 cells, but decreased the growth of tumor cells determined by the reduction of the serum prostate-specific antigen (PSA) [30]. In another study, prostate cancer (LNCaP) cells were injected both intratibially and subcutaneously into eight-week-old SCID mice, followed by administration of OPG [31]. The results of this study displayed that OPG completely prevented the establishment of mixed osteolytic/osteoblastic tibial tumors. In addition, osteoclast numbers were elevated at the bone/tumor interface in the untreated mice whereas OPG-treated mice displayed normal values of osteoclast numbers [31].

Even though OPG has a protective effect against the inhibition of tumor growth progression, the overexpression of OPG is correlated with osteoblastic lesions [32]. Al Nakouzi et al. developed an animal model of prostate cancer using IGR-CaP1 cell lines derived from primary prostate cancer, which was orthotopically injected into six-week-old male athymic nude mice. The animals displayed osteoblastic lesions on the bone structure assessed by high-resolution computed tomographic scans. Mechanistically, the bone microenvironment in the tibia of IGR-CaP1-injected animals showed increased expression of OPG, but not in the control PBS-injected tibias [15]. Both in vitro and in vivo studies conducted by Ye et al. also found that the release of miR-141-3p from prostate cancer cells was transferred to osteoblasts followed by increased expression of OPG via activation of p38 mitogen-activated protein kinase (MAPK) signaling. This condition subsequently promoted osteoblast activity which was conducive to the formation of a bone metastases microenvironment [33]. Yonou et al. evaluated the effects of PSA on the gene and protein expression of OPG and RANKL in human osteoblast-like (MG-63 and SaOS-2) cells. The authors found that PSA stimulated OPG production by osteoblasts. It has been postulated that serine protease activity of PSA promoted the secretion of TGF-β1 leading to the increase in OPG expression. These findings suggested that OPG contributed to the osteoblastic features of prostate cancer bone metastases [34]. An in vitro study by Katopodis et al. found that OPG and Runt-related transcription factor 2 (Runx2) were expressed in both prostate cancer (PC-3) and MG-63 osteoblast-like cells. The co-culture of both cells enhanced OPG expression but did not alter Runx2 expression [35].

On the other hand, the increase in RANKL level is associated with osteolytic lesion [32]. Armstrong et al. conducted an experiment using eight-week-old male CB17 SCID mice injected with prostate cancer (PC3) cells intratibially. The animals experienced PC3-induced osteolytic lesions with tumor burden and increased numbers of osteoclasts at the tumor/bone surface compared to naïve mice 14 days post-injection. In addition, there was a significant increase in systemic and local RANKL expression in tumor-bearing tibias compared to non-tumor-bearing tibias 21 days post-inoculation [36]. An experiment conducted by Whang et al. established a model using eight-week-old SCID mice with intratibial injection of PC-3 cells to produce osteolytic lesions. The results found that subcutaneous administration of a RANKL antagonist (RANK:Fc, 15 mg/kg) effectively blocked the establishment and progression of osteolytic lesions formed by PC-3 cells. In contrast, RANK:Fc treatment did not prevent the formation of osteoblastic lesions but inhibited the progression of established osteoblastic lesions [37].

Taken together, these previous findings reiterate that: (a) OPG may be beneficial in preventing osteolytic lesions but overexpression of OPG leads to osteoblastic lesions, and (b) a high level of RANKL expression causes osteolytic lesions, thus RANKL blockade will potentially limit the formation and progression of osteolytic lesions. Hence, maintenance of a balanced profile between OPG and RANKL may represent a potential therapeutic strategy for interfering with prostate tumor metastases and progression to bone.

### 2.3. The Role of the TGF-β Signaling Axis

Transforming growth factor-beta (TGF-β) is produced by osteoblasts and stored in the mineralized bone matrix in its latent (inactive) form. It is activated during osteoclastic bone resorption to initiate new bone formation by osteoblasts [38]. TGF-β also enhanced the expression of OPG, which inhibits osteoclastogenesis [39]. Coincidentally, the activation of TGF-β also promotes the development of bone metastases via stimulating metastatic tumor cells within bone microenvironment to secrete factors that result in osteolytic destruction of bone [40].

A previous study by Leto et al. investigated the circulating levels of Activin A (a member of the TGF-β superfamily) in prostate cancer patients with or without bone metastases. The results showed that the level of Activin A was significantly higher in prostate cancer patients with bone metastases compared to those without bone metastases, pointing that Activin A might be implicated in the pathogenesis of bone metastases [41]. Another study also indicated that TGF-β2 was secreted from PCa-118b cells (a patient-derived xenograft) generated from the osteoblastic lesion [42]. An animal study done by Mishra et al. emphasized that TGF-β signaling blockade inhibited osteoblastic bone formation and tumor incidence. Four- to five-week-old male athymic nude mice after 10–16 weeks of intracardiac injection with a prostate cancer cell line (PacMetUT1) had osteoblastic bone metastases in the skull, ribs, and femur [43]. Knockdown of TGF-β1 in mice and systemic administration of TGF-β1 receptor kinase inhibitor were found to decrease bone tumor growth and osteoblastic bone formation in vivo after seven weeks [43]. Additionally, Rafiei and Komarova reported that inhibition of TGF-β receptor 1 and macrophage-colony stimulating factors (M-CSF) synergistically resulted in attenuation of prostate cancer-induced osteoclastogenesis [44].

On the other hand, other studies have reported contrary outcomes on the role of TGF-β in prostate cancer bone metastases. An in vitro study by AlShaibi et al. found that the TGF-β derived from prostate cancer cells induced the expression of Noggin, which is an important suppressor of the differentiation of osteoblast lineage cells in bone metastases [45]. Whereas findings from a study by Katopodis et al. showed that the enhancement of OPG expression in PC-3 cells by MG-63 cells is not mediated by TGF-β1 [35]. Hence, findings from these studies implied that TGF-β has complex and divergent roles in bone homeostasis and the dysregulation of the TGF-β signaling axis has implications in bone disease.

### 2.4. The Role of Bone Morphogenetic Protein (BMP)

Bone morphogenetic protein (BMP) belongs to the TGF-β superfamily, which functionally stimulates the replication and differentiation of normal cells in the osteoblast lineage. It also plays a crucial role during the process of mesoderm induction, neural tissue differentiation, and morphogenesis of various tissues [39,46]. Interestingly, BMPs are not only synthesized by osteoblasts but also secreted by prostate cancers. The unusual expression of BMPs in prostate cancer has been implicated in the progression of the disease.

A study by Bobinac et al. investigated the expression of BMP-2, BMP-3, BMP-4, BMP-5, BMP-6, and BMP-7 in cancer tissue obtained from prostate cancer patients with established bone metastases. The results showed that all BMPs were expressed in all malignant and normal prostate tissues. Specifically, the expression of BMP-3 and BMP-5 was relatively higher whereas the expression of BMP-7 was comparatively lower in prostate cancer tissue than normal tissue. However, the expression of other BMPs such as BMP-2/4 and BMP-6 was not significantly different. The authors confirmed that different types of BMPs displayed different expression levels, thus identifying that BMP proteins might be useful for monitoring tumor status in prostate cancer with bone metastases [47]. Another study by Feeley et al. demonstrated that: (a) High BMP receptors were expressed in the PC-3 cells; (b) BMP-2 stimulated PC-3 cell proliferation; (c) BMP-2 and BMP-4 stimulated PC-3 cell migration and invasion; and (d) BMP-7 had no effect on PC-3 cell proliferation, migration, or invasion. In the same study, PC-3 cells implanted into SCID mouse tibia resulted in the formation of osteolytic lesions as early as two weeks and completely destroyed the proximal tibia at week eight. This study suggested that BMPs might influence the formation of osteolytic prostate cancer metastases [48].

Autzen et al. also examined the expression of BMP-6 mRNA in matched prostatic primary and secondary bony lesions and in isolated skeletal metastases from prostatic adenocarcinomas. They found that BMP-6 mRNA was detected in 11 out of 13 bone metastases from samples of prostate carcinoma patients. The BMP-6 mRNA appeared to be strongly expressed in prostatic adenocarcinoma both in the primary tumor and in bone metastases [49]. Masuda et al. have investigated the biological relationship between the expressions of BMP-6 and BMP-7 in normal and metastatic bone tissues in an earlier study. This study revealed that the expression level of BMP-7 was significantly higher in metastatic bone lesions than in normal bone. However, there was no significant difference between the level of BMP-6 expression in metastatic bone lesions from prostate cancer and the level in normal bone tissue [50]. A further study done by Masuda and colleagues found a high expression level of BMP-7 in the normal prostate glandular tissues while BMP-7 expression was low during the development and progression of prostate cancer. In the same study, they also examined the expression of BMP-7 in human prostatic epithelial cells under androgen replacement and the results showed higher BMP-7 expression in the dihydrotestosterone-treated prostate epithelial cells than non-treated groups [51].

The Wingless (Wnt) proteins, produced by prostate cancer, have been shown to have autocrine tumor effects through enhancing the proliferation of the tissue. Interestingly, it can also act as a paracrine hormone to induce osteoblastic activity in bone metastases [10]. The ability of BMPs and Wnts as mediators to regulate osteoblastic activity in prostate cancer bone metastases have been evaluated by Dai et al. Administration of Wnt3a and Wnt5a, or knockdown of DKK-1 (a Wnt inhibitor), induced BMP-4 and BMP-6 expressions and promoted activation in prostate cancer cells. Wnt3a, Wnt5a, and conditioned medium from C4-2B or LuCaP 23.1 cells have been identified to induce osteoblast differentiation in vitro. With the addition of DKK-1 and Noggin (antagonist of BMPs) to the conditioned medium, the activity of prostate cancer-induced osteoblast differentiation was diminished. Results also found that knockdown of BMP expression in C4-2B cells inhibited Wnt-induced osteoblastic activity. These findings indicated that Wnts and BMPs have a strong relationship in prostate cancer-induced osteoblast differentiation [52].

Taken together, BMP expressions are detectable in either normal prostate tissue or prostate cancer cells. The pattern of BMP expression has a close relationship with the progression of prostate cancer and contributes to the onset of bone lesions. It is clear that BMPs play a role in the vicious cycle of metastatic bone formation from prostate cancer. BMPs produced by prostate cancer will induce osteoblastic activities and promote osteoblastic lesions. On the other hand, BMPs synthesized by osteoblasts subsequently enhance the growth of prostate cancer cells allowing further production of BMPs from prostate cancer.

### 2.5. The Role of Other Growth Factors

The involvement of other growth factors and their respective receptors in metastasis of prostate cancer has been extensively investigated, as they are believed to enhance the invasiveness of prostate cancer. So far, the growth factors and growth factor receptors tested were growth differentiation factor 15 (GDF15), fibroblast growth factor 3, 9, and 19 (FGF3, FGF9, and FGF19), chemokine C-X-C motif ligand 1 (CXCL1), galectins, β2-microglobulin, IGF-1, IGF-2, the epidermal growth factor receptor (EGFR), the hepatocyte growth factor receptor (HGFR), as well as the vascular endothelial growth factor receptor 2 (VEGFR2).

An extensive study by Lee et al. studied various growth factors (including GDF15, FGF3, FGF19, CXCL1, galectins, and β2-microglobulin) critical to the tumor-bone-microenvironment events in a patient-derived xenograft (PCa-118b) generated from osteoblastic bone lesions. Researchers have identified the interplay of these secretory proteins that exerted both autocrine and paracrine influence on tumor and stromal cells (osteoblasts) [42]. The role of FGF9 and AR in the mechanism of prostate cancer pathogenesis, in view of its resistance to castration, has been investigated [53]. FGF9 induced osteoblast proliferation and new bone formation in human AR-negative prostate cancer xenografts which overexpressed FGF9 relative to other bone-derived prostate cancer cells. Positive FGF9 immunostaining in prostate cancer cells was recorded in all (100%) cases of bone metastasis as compared to 43% in primary tumors derived from human organ-confined prostate cancer. Furthermore, mice treated with FGF9-neutralizing antibody were seen to develop smaller bone tumors and reduced bone formation [53]. Moreover, the effects of bone-associated growth factors such as IGF-1 and IGF-2 on prostate cancer cell proliferation were evaluated. These growth factors produced by bone cells increased proliferation of three different prostate carcinoma cell lines (LNCaP, PC-3, and DU-145) [54].

Apart from that, the role of EGFR/Erb-B2 receptor tyrosine kinase 2 (ERBB2) signaling in prostate cancer metastasis into the bone microenvironment has also been enumerated [55]. The study suggested that osteoblast-directed induction of signaling activity involving EGFR and ERBB2 in prostate carcinoma cells might be culpable in bone metastasis. EGFR and ERBB2 activation in LNCaP cells under the influence of osteoblast-derived sarcoma cells (OHS) has been reported to activate EGFR/ERBB2 signaling pathways in LNCaP cells co-cultured with osteoblastic cells that had been differentiated from human mesenchymal stem cells or OHS cells. This finding supported the rationale for the use of EGFR or ERBB2 inhibitors for prophylaxis or cure of prostate cancer metastasis in the androgen-sensitive stage [55]. 

A study demonstrated that dual-kinase inhibition of c-Met (a HGFR) and VEGFR2 by cabozantinib reduced cancer growth in bone and inhibited osteoblasts in models using the prostate cancer cells PC-3 and C4-2B [56]. Although the signaling mechanism remains unclear, inhibition of c-Met and/or VEGFR2 was able to reverse osteoclastogenesis promoted by conditioned media from osteoblasts treated with IGF-1, hepatocyte growth factor (HGF), or vascular endothelial growth factor-A (VEGF-A). Furthermore, HGFR and VEGFR2 inhibition caused a reduction in RANKL and M-CSF, which are the factors essential for osteoclastogenesis [56].

In summary, the accumulated evidence postulated that growth factors are signaling molecules, which support the events involving the tumor and the bone microenvironment during prostate cancer metastases. These growth factors directly increased tumor cell proliferation as well as engage bone stromal cells via stimulation of osteogenesis and osteoclastogenesis (bone matrix turnover), thus promoting metastatic activities.

### 2.6. The Role of Inflammation

Acute inflammation is a biological response triggered by harmful stimuli such as infection, trauma, and tissue injury to eliminate the source of damage [46]. The tumor microenvironment is unequivocally linked with inflammation, whether the infiltration of immune cells engages with tumor cells causing inflammation or chronic inflammation promotes the malignant transformation of cells and carcinogenesis [57,58].

A study by Lu et al. measured the cytokine expression in conditioned medium collected from primary prostate epithelial cells (PrEC) and prostate cancer cells (PC-3) cells. Results obtained from this study found that prostate cancer cells produced a high amount of monocyte chemotactic protein-1 (MCP-1) and interleukin-8 (IL-8) compared to PrEC cells. It also found that neutralizing antibodies for MCP-1 and IL-8 synergistically inhibited the prostate cancer conditioned, medium-induced bone resorption [59]. In a subsequent study, Lu et al. reported that activation of the MCP-1/CCR2 axis promoted prostate cancer growth in bone. Firstly, the authors found elevated MCP-1 level in the serum of patients with bone metastases compared to localized prostate cancer. Secondly, CCR2 knockdown significantly diminished the MCP-1-induced prostate cancer cell invasion. Thirdly, MCP-1 knockdown significantly decreased prostate cancer conditioned, medium-induced osteoclast formation. Finally, an in vivo study found that MCP-1 knockdown PC-3 cells implanted into the tibia of SCID mice for four weeks diminished PC-3 tumor growth in bone [60].

In an experiment performed by Morrissey et al., it was found that IL-6 was highly expressed in prostate cancer bone metastases. PC-3 cells inhibited osteoblast activity and induced osteoblast to produce IL-6 that promoted osteoclastogenesis [61]. In addition, a recent study by Roca et al. observed that macrophage-driven efferocytosis (a process of continually clearing of apoptotic cancer cells by immune system phagocytes during tumor progression) induced the expression of pro-inflammatory cytokines, such as C-X-C motif chemokine ligand 5 (CXCL5) by activating the signal transducer and activator of transcription 3 (STAT3) and the nuclear factor kappa-light-chain-enhancer of activated B cells (NF-κB) signaling. CXCL5-deficient mice had reduced tumor progression. These findings suggested that the myeloid phagocytic clearance of apoptotic cancer cells accelerated CXCL5-mediated inflammation and tumor growth in bone [62]. In summary, findings from available evidence suggest the alleviation of chronic inflammation as a potential therapeutic approach for prostate cancer bone metastases.

### 2.7. The Role of Biochemical Markers

The involvement of endogenous biochemical markers of bone metabolism in cancer metastasis into the bone microenvironment has been a subject of scrutiny for decades. Bone metastases from prostate carcinoma have been characterized as predominantly osteoblastic and/or osteolytic in nature. In view of this, the serum level of bone-specific ALP, a marker of osteoblast proliferation, is evidently elevated during the course of bone metastases [8,63]. Other studies on markers of bone metabolism such as osteocalcin (OCN), urinary calcium, hydroxyproline, and pyridinoline have been carried out [23,24,64]. On the other hand, both pyridinoline and deoxypyridinoline (bone resorption markers) were significantly elevated in prostate cancer patients with bone metastasis when compared to control group and patients without bone metastasis [65]. The authors suggested that these bone markers have a good degree of bone specificity by reflecting the extent of bone metastasis more accurately than other bone markers and PSA due to their bone-specificity as they are not metabolized internally and unaffected by diet [65]. In a study involving 83 specimens from 70 patients with prostatic cancer (32 with and 38 without bone metastasis), the pyridinoline cross-linked carboxyterminal telopeptide (CTP) level was correlated with the extent of disease score more than PSA and other bone markers [64]. In another study, the level of TRAP (a bone resorption marker) and total regional bone mineral content (BMC) measured in 64 patients (32 patients and 32 controls) was found to be significantly different in the pelvis, legs, and trunk of patients compared to the control [66]. These findings confirmed a corresponding increase in bone resorptive (osteoclastic) activity alongside bone osteoblastic activity during bone metastasis [64,65].

Urokinase, a member of serine proteases that also function as a growth factor to osteoblastic cells, is believed to play a catalytic role in the metastasis of prostate cancer to the skeleton and extraskeletal sites. In a model of urokinase overexpression, Copenhagen rats inoculated with MatLyLu rat prostate carcinoma cells transfected with plasmids encoding overexpression of urokinase (pYN-ruPA, pYN-ruPA-AS) were investigated for the pattern of metastasis [67]. The study outcomes revealed significantly earlier and more widespread development of bone metastasis in the ribs, scapula, and femora of rats inoculated with pYN-ruPA (14–15 days) as compared to the control that manifested metastasis only in the lumbar vertebrae at 20–21 days post inoculation. Biochemical assay and histological evaluation revealed an accompanying progressive increase in serum ALP level and osteoblastic activity compared to the control animals [67].

Another marker, p45-sErbB3 (belonging to the ErbB3 family), has been shown to play a vital role in bone metastasis of prostate cancer. In a clinical study involving 108 men with androgen-dependent and independent disease, higher levels of sErbB3 were seen in men with a positive and negative bone scan [68]. However, men with the androgen-independent disease and a positive bone scan with higher sErbB3 took longer time to show detectable bone metastases (82 months) than in men with lower sErbB3 (41 months). Although high levels of sErbB3 did not imply better survival benefit after bone metastasis, the correlation between higher sErbB3 levels and longer bone metastasis time indicated that sErbB3 had a role in prostate cancer metastasis in bone [68].

In a bid to understand the mechanism of prostate cancer-induced osteoblastic differentiation, semaphorin 3A (Sema 3A), a marker that is believed to be involved in the regulation of bone metabolism, has been investigated [69]. In a study utilizing cultured osteoprogenitor MC3T3-E1 cells, three prostate cancer cell lines (C4–2, LNCaP, and PC-3) and Sema 3A short hairpin RNA, Western blot and immunofluorescence analysis revealed higher Sema 3A production in C4-2 cells than in LNCaP and PC-3 cells. Down-regulation of Sema 3A expression caused a corresponding decrease in C4-2 cell lines, which implied the inhibition of osteogenic differentiation manifested by decreased ALP and osteoblastic mineralization. Furthermore, neutralizing Sema 3A in C4-2 cells with Sema 3A short hairpin RNA diminished the expression of β-catenin in the MC3T3-E1 cells. Thus, C4-2 cell-induced osteogenic differentiation in MC3T3-E1 cells was believed to be mediated by Sema 3A/NRP1 signaling.

### 2.8. The Role of Estrogen

The presence of an estrogen receptor in the prostate suggests that estrogen may act directly in the prostate epithelial cells [40]. An earlier in vivo study demonstrated that the combination of estradiol and testosterone increased the incidence of prostate cancer to nearly 100% [70]. Functional estrogen receptor-alpha (ERα) is essential for the development of prostate cancer, evidenced by the inability of testosterone and estradiol to induce prostate cancer in ERα-knockout mice [71]. Apart from that, estrogen is found to exert agonistic effects on bone tissue. Inevitably, the direct action of estrogen on bone cells (osteoblast, osteoclast, and osteocytes) results in increased bone formation and reduced bone resorption [72]. The lack of ERα-mediated RANKL suppression contributed to the increase in bone resorption in the estrogen-deficient mice [73]. Since estrogen and ERα signaling are the common factors for bone metastasis and bone remodeling, the potential role of estrogen in prostate cancer bone metastasis is considered.

Evidence from Mishra et al. found that prostate cancer PacMetUT1 cells with ERα-knockdown were able to inhibit osteoblastic lesion formation [74]. The results also suggested that estrogen signaling promoted crosstalk between cancer and osteoblastic progenitors to stimulate osteoblastic tumorigenesis through significant induction of osteogenic markers in pre-osteoblast co-cultured with cancer cells. Thus, inhibition of ERα signaling in prostate cancer cells may be beneficial in order to inhibit osteoblastic lesion development, especially in patients with prostate cancer.

## 3. Conclusions

**Figure 1:**
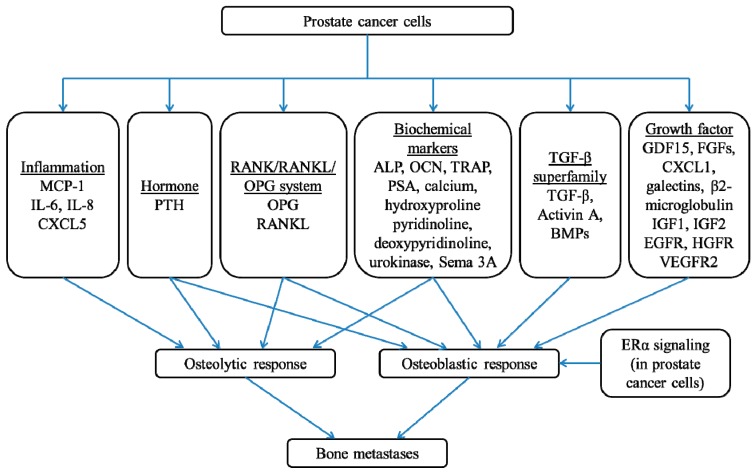
The signaling molecules involved in governing bone metastases driven by prostate cancer.

Prostate cancer cells have a strong predilection to spread to the bone, promoting osteolytic and/or osteoblastic lesions via various signaling molecules aforementioned (Figure 1). Once bone metastasis is established, the current treatment is designed to be palliative and not curative which aims to decrease tumor burden, prevent further progression and metastasis of tumor cells, and alleviate tumor-associated bone pathologies (such as fracture and pain) [75]. Thus, the effort in searching for a potential therapeutic intervention deterring the metastasis of prostate carcinoma to the bone can be very challenging. Following tumor expansion to the metastatic stage, the therapeutic strategies should not only focus on inducing cancer cell apoptosis, but also targeting the survival factors and their subsequent signaling networks critical for the progression of bone metastases.

Currently, there are a few therapeutic agents that have been approved by the Food and Drug Administration (FDA) to prevent skeletal-related events in metastatic castration-resistant prostate cancer, such as bisphosphonate (zoledronic acid) and denosumab. Both of these agents showed their ability to reduce bone fragility [76,77,78] and delayed metastases [79]. Interestingly, new agents in prostate cancer with specific effects on bone like cabozantinib (XL184) [80] and dasatinib [81], which acted as a novel receptor tyrosine kinase inhibitor, have also been investigated. Another potential agent that can be used in prostate cancer treatment is endothelin-A receptor (ETAR) antagonist, such as atrasentan. Endothelin-1 (ET-1) is known to have a role in the pathogenesis of cancer, particularly prostate cancer. It acts through its receptor, ETAR, to promote cell proliferation [82]. A meta-analysis conducted by Qiao et al. reported that treatment with atrasentan lowered the level of PSA and incidence of bone pain. Besides, the increasing of PSA and bone ALP were delayed in atrasentan-treated prostate cancer patients. These outcomes reiterated that atrasentan exerted a potential effect in controlling cancer-related bone pain and skeletal complications in prostate cancer patients [83].

This review has uncovered the multifactorial mechanisms and complex tumor-bone interactions that occur in the bone metastatic microenvironment. We justify that the modulation of bone resorption and bone formation is the main concept for inhibition of bone metastases since the bone microenvironment contributes to the aggressive behavior of metastatic prostate cancer cells. The decrease in bone resorption and increase in bone formation will potentially improve an osteolytic lesion, while the decrease in bone formation and increase in bone resorption will potentially improve an osteoblastic lesion. However, the concomitant occurrence of osteoblastic bone lesions along with osteolytic bone lesions in advanced prostate cancer further complicates the conditions and prevents the development of a bone-directed therapy.

## Abbreviations

ALP	Alkaline phosphatase
AR	Androgen receptor
BMC	Bone mineral content
BMD	Bone mineral density
BMP	Bone morphogenetic protein
CTP	Pyridinoline cross-linked carboxyterminal telopeptide
CXCL1	C-X-C motif chemokine ligand 1
CXCL5	C-X-C motif chemokine ligand 5
EGFR	Epidermal growth factor receptor
ERα	Estrogen receptor-alpha
ERBB2	Erb-B2 receptor tyrosine kinase 2
ET-1	Endothelin-1
ETAR	Endothelin-A receptor
FDA	Food and Drug Administration
FGF	Fibroblast growth factor
GDF15	Growth differentiation factor 15
HGF	Hepatocyte growth factor
HGFR	Hepatocyte growth factor receptor
IGF	Insulin-like growth factor
IL-8	Interleukin-8
MAPK	Mitogen-activated protein kinase
MCP-1	Monocyte chemotactic protein-1
M-CSF	Macrophage-colony stimulating factors
NF-κB	Nuclear factor kappa-light-chain-enhancer of activated B cells
OCN	Osteocalcin
OHS	Osteoblast-derived sarcoma cells
OPG	Osteoprotegerin
PSA	Prostate-specific antigen
PTH	Parathyroid hormone
PTHrP	Parathyroid hormone-related peptide
RANK	Receptor activator of nuclear factor-kappa B
RANKL	Receptor activator of nuclear factor-kappa B ligand
Runx2	Runt-related transcription factor 2
SCID	Severe combined immunodeficient
Sema 3A	Semaphorin 3A
STAT3	Signal transducer and activator of transcription 3
TGF-β	Transforming growth factor-beta
TRAP	Tartrate-resistant acid phosphatase
VEGF-A	Vascular endothelial growth factor-A
VEGFR2	Vascular endothelial growth factor receptor 2
Wnt	Wingless

## References

[1] Baade P.D., Youlden D.R., Cramb S.M., Dunn J., Gardiner R.A. (2013). Epidemiology of prostate cancer in the Asia-Pacific region. Prostate Int..

[2] Bubendorf L., Schöpfer A., Wagner U., Sauter G., Moch H., Willi N., Gasser T.C., Mihatsch M.J. (2000). Metastatic patterns of prostate cancer: An autopsy study of 1,589 patients. Hum. Pathol..

[3] Paget S. (1889). The distribution of secondary growths in cancer of the breast. Lancet.

[4] Bagi C. (2003). Skeletal implications of prostate cancer. J. Musculoskelet. Neuronal. Interact.

[5] Kingsley L.A., Fournier P.G., Chirgwin J.M., Guise T.A. (2007). Molecular biology of bone metastasis. Mol Cancer Ther..

[6] Yin J.J., Pollock C.B., Kelly K. (2005). Mechanisms of cancer metastasis to the bone. Cell Res..

[7] Westendorf J.J., Kahler R.A., Schroeder T.M. (2004). Wnt signaling in osteoblasts and bone diseases. Gene.

[8] Roodman G.D. (2004). Mechanisms of bone metastasis. N. Engl. J. Med..

[9] Abou-Samra A.B., Juppner H., Force T., Freeman M.W., Kong X.F., Schipani E., Urena P., Richards J., Bonventre J.V., Potts J.T. (1992). Expression cloning of a common receptor for parathyroid hormone and parathyroid hormone-related peptide from rat osteoblast-like cells: A single receptor stimulates intracellular accumulation of both cAMP and inositol trisphosphates and increases intracellular free calcium. Proc. Natl. Acad. Sci. USA.

[10] Hall C.L., Kang S., MacDougald O.A., Keller E.T. (2006). Role of Wnts in prostate cancer bone metastases. J. Cell Biochem..

[11] Ellis W.J., Vessella R.L., Buhler K.R., Bladou F., True L.D., Bigler S.A., Curtis D., Lange P.H. (1996). Characterization of a novel androgen-sensitive, prostate-specific antigen-producing prostatic carcinoma xenograft: LuCaP 23. Clin. Cancer Res..

[12] Lim D.J., Liu X.L., Sutkowski D.M., Braun E.J., Lee C., Kozlowski J.M. (1993). Growth of an androgen-sensitive human prostate cancer cell line, LNCaP, in nude mice. Prostate.

[13] Liu A.Y., Brubaker K.D., Goo Y.A., Quinn J.E., Kral S., Sorensen C.M., Vessella R.L., Belldegrun A.S., Hood L.E. (2004). Lineage relationship between LNCaP and LNCaP-derived prostate cancer cell lines. Prostate.

[14] Li T.-H., Zhao H., Peng Y., Beliakoff J., Brooks J.D., Sun Z. (2007). A promoting role of androgen receptor in androgen-sensitive and -insensitive prostate cancer cells. Nucleic Acids Res..

[15] Al Nakouzi N., Bawa O., Le Pape A., Lerondel S., Gaudin C., Opolon P., Gonin P., Fizazi K., Chauchereau A. (2012). The IGR-CaP1 xenograft model recapitulates mixed osteolytic/blastic bone lesions observed in metastatic prostate cancer. Neoplasia.

[16] Huang J.C., Sakata T., Pfleger L.L., Bencsik M., Halloran B.P., Bikle D.D., Nissenson R.A. (2004). PTH differentially regulates expression of RANKL and OPG. J. Bone Miner Res..

[17] Kandil E., Noureldine S., Khalek M.A., Daroca P., Friedlander P. (2011). Ectopic secretion of parathyroid hormone in a neuroendocrine tumor: A case report and review of the literature. Int. J. Clin. Exp. Med..

[18] Demura M., Yoneda T., Wang F., Zen Y., Karashima S., Zhu A., Cheng Y., Yamagishi M., Takeda Y. (2010). Ectopic production of parathyroid hormone in a patient with sporadic medullary thyroid cancer. Endocr. J..

[19] Nussbaum S.R., Gaz R.D., Arnold A. (1990). Hypercalcemia and ectopic secretion of parathyroid hormone by an ovarian carcinoma with rearrangement of the gene for parathyroid hormone. N. Engl. J. Med..

[20] Yoshimoto K., Yamasaki R., Sakai H., Tezuka U., Takahashi M., Iizuka M., Sekiya T., Saito S. (1989). Ectopic production of parathyroid hormone by small cell lung cancer in a patient with hypercalcemia. J. Clin. Endocrinol. Metab..

[21] Strewler G.J., Budayr A.A., Clark O.H., Nissenson R.A. (1993). Production of parathyroid hormone by a malignant nonparathyroid tumor in a hypercalcemic patient. J. Clin. Endocrinol. Metab..

[22] Schwartz G.G. (2008). Prostate cancer, serum parathyroid hormone, and the progression of skeletal metastases. Cancer Epidemiol. Biomark. Prev..

[23] Cooper E., Whelan P., Purves D. (1994). Bone alkaline phosphatase and prostate-specific antigen in the monitoring of prostate cancer. Prostate.

[24] Kylmälä T., Tammela T., Risteli L., Risteli J., Kontturi M., Elomaa I. (1995). Type I collagen degradation product (ICTP) gives information about the nature of bone metastases and has prognostic value in prostate cancer. Br. J. Cancer.

[25] Philbrick W., Wysolmerski J., Galbraith S., Holt E., Orloff J., Yang K., Vasavada R., Weir E., Broadus A., Stewart A. (1996). Defining the roles of parathyroid hormone-related protein in normal physiology. Physiol. Rev..

[26] Blomme E.A., Dougherty K.M., Pienta K.J., Capen C.C., Rosol T.J., McCauley L.K. (1999). Skeletal metastasis of prostate adenocarcinoma in rats: Morphometric analysis and role of parathyroid hormone-related protein. Prostate.

[27] Rabbani S.A., Gladu J., Harakidas P., Jamison B., Goltzman D. (1999). Over-production of parathyroid hormone-related peptide results in increased osteolytic skeletal metastasis by prostate cancer cells in vivo. Int. J. Cancer.

[28] Liao J., Li X., Koh A.J., Berry J.E., Thudi N., Rosol T.J., Pienta K.J., McCauley L.K. (2008). Tumor expressed PTHrP facilitates prostate cancer-induced osteoblastic lesions. Int. J. Cancer.

[29] Corey E., Brown L.G., Kiefer J.A., Quinn J.E., Pitts T.E., Blair J.M., Vessella R.L. (2005). Osteoprotegerin in prostate cancer bone metastasis. Cancer Res..

[30] Kiefer J.A., Vessella R.L., Quinn J.E., Odman A.M., Zhang J., Keller E.T., Kostenuik P.J., Dunstan C.R., Corey E. (2004). The effect of osteoprotegerin administration on the intra-tibial growth of the osteoblastic LuCaP 23.1 prostate cancer xenograft. Clin. Exp. Metast..

[31] Zhang J., Dai J., Qi Y., Lin D.L., Smith P., Strayhorn C., Mizokami A., Fu Z., Westman J., Keller E.T. (2001). Osteoprotegerin inhibits prostate cancer-induced osteoclastogenesis and prevents prostate tumor growth in the bone. J. Clin. Investig..

[32] Powell G.J., Southby J., Danks J.A., Stillwell R.G., Hayman J.A., Henderson M.A., Bennett R.C., Martin T.J. (1991). Localization of parathyroid hormone-related protein in breast cancer metastases: Increased incidence in bone compared with other sites. Cancer Res..

[33] Ye Y., Li S.L., Ma Y.Y., Diao Y.J., Yang L., Su M.Q., Li Z., Ji Y., Wang J., Lei L. (2017). Exosomal miR-141-3p regulates osteoblast activity to promote the osteoblastic metastasis of prostate cancer. Proc. Natl. Acad. Sci. USA.

[34] Yonou H., Horiguchi Y., Ohno Y., Namiki K., Yoshioka K., Ohori M., Hatano T., Tachibana M. (2007). Prostate-specific antigen stimulates osteoprotegerin production and inhibits receptor activator of nuclear factor-kappaB ligand expression by human osteoblasts. Prostate.

[35] Katopodis H., Philippou A., Tenta R., Doillon C., Papachroni K.K., Papavassiliou A.G., Koutsilieris M. (2009). MG-63 osteoblast-like cells enhance the osteoprotegerin expression of PC-3 prostate cancer cells. Anticancer Res..

[36] Armstrong A.P., Miller R.E., Jones J.C., Zhang J., Keller E.T., Dougall W.C. (2008). RANKL acts directly on RANK-expressing prostate tumor cells and mediates migration and expression of tumor metastasis genes. Prostate.

[37] Whang P.G., Schwarz E.M., Gamradt S.C., Dougall W.C., Lieberman J.R. (2005). The effects of RANK blockade and osteoclast depletion in a model of pure osteoblastic prostate cancer metastasis in bone. J. Orthop. Res..

[38] Barrow J.R., Thomas K.R., Boussadia-Zahui O., Moore R., Kemler R., Capecchi M.R., McMahon A.P. (2003). Ectodermal Wnt3/β-catenin signaling is required for the establishment and maintenance of the apical ectodermal ridge. Genes Dev..

[39] Martinovic S., Borovecki F., Sampath K.T., Vukicevic S. (2002). Biology of bone morphogenetic proteins. Bone Morphogenetic Proteins.

[40] Prins G.S., Korach K.S. (2008). The role of estrogens and estrogen receptors in normal prostate growth and disease. Steroids.

[41] Leto G., Incorvaia L., Badalamenti G., Tumminello F.M., Gebbia N., Flandina C., Crescimanno M., Rini G. (2006). Activin A circulating levels in patients with bone metastasis from breast or prostate cancer. Clin. Exp. Metast..

[42] Lee Y.C., Gajdosik M.S., Josic D., Clifton J.G., Logothetis C., Yu-Lee L.Y., Gallick G.E., Maity S.N., Lin S.H. (2015). Secretome analysis of an osteogenic prostate tumor identifies complex signaling networks mediating cross-talk of cancer and stromal cells within the tumor microenvironment. Mol. Cell Proteom..

[43] Mishra S., Tang Y., Wang L., de Graffenried L., Yeh I.T., Werner S., Troyer D., Copland J.A., Sun L.Z. (2011). Blockade of transforming growth factor-beta (TGFβ) signaling inhibits osteoblastic tumorigenesis by a novel human prostate cancer cell line. Prostate.

[44] Rafiei S., Komarova S.V. (2013). Molecular signaling pathways mediating osteoclastogenesis induced by prostate cancer cells. BMC Cancer.

[45] AlShaibi H.F., Ahmed F., Buckle C., Fowles A.C.M., Awlia J., Cecchini M.G., Eaton C.L. (2017). The BMP antagonist Noggin is produced by osteoblasts in response to the presence of prostate cancer cells. Biotechnol. Appl. Biochem..

[46] Hogan B. (1996). Bone morphogenetic proteins: Multifunctional regulators of vertebrate development. Genes Dev..

[47] Bobinac D., Maric I., Zoricic S., Spanjol J., Dordevic G., Mustac E., Fuckar Z. (2005). Expression of bone morphogenetic proteins in human metastatic prostate and breast cancer. Croat Med. J..

[48] Feeley B.T., Krenek L., Liu N., Hsu W.K., Gamradt S.C., Schwarz E.M., Huard J., Lieberman J.R. (2006). Overexpression of noggin inhibits BMP-mediated growth of osteolytic prostate cancer lesions. Bone.

[49] Autzen P., Robson C.N., Bjartell A., Malcolm A.J., Johnson M.I., Neal D.E., Hamdy F.C. (1998). Bone morphogenetic protein 6 in skeletal metastases from prostate cancer and other common human malignancies. Br. J. Cancer.

[50] Masuda H., Fukabori Y., Nakano K., Takezawa Y., T C.S., Yamanaka H. (2003). Increased expression of bone morphogenetic protein-7 in bone metastatic prostate cancer. Prostate.

[51] Masuda H., Fukabori Y., Nakano K., Shimizu N., Yamanaka H. (2004). Expression of bone morphogenetic protein-7 (BMP-7) in human prostate. Prostate.

[52] Dai J., Hall C.L., Escara-Wilke J., Mizokami A., Keller J.M., Keller E.T. (2008). Prostate cancer induces bone metastasis through Wnt-induced bone morphogenetic protein-dependent and independent mechanisms. Cancer Res..

[53] Li Z.G., Mathew P., Yang J., Starbuck M.W., Zurita A.J., Liu J., Sikes C., Multani A.S., Efstathiou E., Lopez A. (2008). Androgen receptor-negative human prostate cancer cells induce osteogenesis in mice through FGF9-mediated mechanisms. J. Clin. Investig..

[54] Ritchie C.K., Andrews L.R., Thomas K.G., Tindall D.J., Fitzpatrick L.A. (1997). The effects of growth factors associated with osteoblasts on prostate carcinoma proliferation and chemotaxis: Implications for the development of metastatic disease. Endocrinology.

[55] Bratland A., Boender P.J., Hoifodt H.K., Ostensen I.H., Ruijtenbeek R., Wang M.Y., Berg J.P., Lilleby W., Fodstad O., Ree A.H. (2009). Osteoblast-induced EGFR/ERBB2 signaling in androgen-sensitive prostate carcinoma cells characterized by multiplex kinase activity profiling. Clin. Exp. Metast..

[56] Lee C., Whang Y.M., Campbell P., Mulcrone P.L., Elefteriou F., Cho S.W., Park S.I. (2018). Dual targeting c-met and VEGFR2 in osteoblasts suppresses growth and osteolysis of prostate cancer bone metastasis. Cancer Lett..

[57] Azrina A., Khoo H.E., Idris M.A., Amin I., Razman M.R. (2011). Major inorganic elements in tap water samples in Peninsular Malaysia. Malays. J. Nutr..

[58] Wheater G., Elshahaly M., Tuck S.P., Datta H.K., van Laar J.M. (2013). The clinical utility of bone marker measurements in osteoporosis. J. Transl. Med..

[59] Lu Y., Cai Z., Xiao G., Keller E.T., Mizokami A., Yao Z., Roodman G.D., Zhang J. (2007). Monocyte chemotactic protein-1 mediates prostate cancer-induced bone resorption. Cancer Res..

[60] Lu Y., Chen Q., Corey E., Xie W., Fan J., Mizokami A., Zhang J. (2009). Activation of MCP-1/CCR2 axis promotes prostate cancer growth in bone. Clin. Exp. Metast..

[61] Morrissey C., Lai J.S., Brown L.G., Wang Y.C., Roudier M.P., Coleman I.M., Gulati R., Vakar-Lopez F., True L.D., Corey E. (2010). The expression of osteoclastogenesis-associated factors and osteoblast response to osteolytic prostate cancer cells. Prostate.

[62] Southby J., Kissin M.W., Danks J.A., Hayman J.A., Moseley J.M., Henderson M.A., Bennett R.C., Martin T.J. (1990). Immunohistochemical localization of parathyroid hormone-related protein in human breast cancer. Cancer Res..

[63] Logothetis C.J., Lin S.H. (2005). Osteoblasts in prostate cancer metastasis to bone. Nat. Rev. Cancer.

[64] Maeda H., Koizumi M., Yoshimura K., Yamauchi T., Kawai T., Ogata E. (1997). Correlation between bone metabolic markers and bone scan in prostatic cancer. J. Urol..

[65] Sano M., Kushida K., Takahashi M., Ohishi T., Kawana K., Okada M., Inoue T. (1994). Urinary pyridinoline and deoxypyridinoline in prostate carcinoma patients with bone metastasis. Br. J. Cancer.

[66] Revilla M., Arribas I., Sanchez-Chapado M., Villa L.F., Bethencourt F., Rico H. (1998). Total and regional bone mass and biochemical markers of bone remodeling in metastatic prostate cancer. Prostate.

[67] Achbarou A., Kaiser S., Tremblay G., Ste-Marie L.G., Brodt P., Goltzman D., Rabbani S.A. (1994). Urokinase overproduction results in increased skeletal metastasis by prostate cancer cells in vivo. Cancer Res..

[68] Lin S.H., Lee Y.C., Choueiri M.B., Wen S., Mathew P., Ye X., Do K.A., Navone N.M., Kim J., Tu S.M. (2008). Soluble ErbB3 levels in bone marrow and plasma of men with prostate cancer. Clin. Cancer Res..

[69] Liu F., Shen W., Qiu H., Hu X., Zhang C., Chu T. (2015). Prostate cancer cells induce osteoblastic differentiation via semaphorin 3A. Prostate.

[70] Bosland M.C., Ford H., Horton L. (1995). Induction at high incidence of ductal prostate adenocarcinomas in NBL/Cr and Sprague-Dawley Hsd:SD rats treated with a combination of testosterone and estradiol-17 beta or diethylstilbestrol. Carcinogenesis.

[71] Ricke W.A., McPherson S.J., Bianco J.J., Cunha G.R., Wang Y., Risbridger G.P. (2008). Prostatic hormonal carcinogenesis is mediated by in situ estrogen production and estrogen receptor alpha signaling. FASEB J..

[72] Khosla S., Oursler M.J., Monroe D.G. (2012). Estrogen and the skeleton. Trends Endocrinol. Metab..

[73] Streicher C., Heyny A., Andrukhova O., Haigl B., Slavic S., Schüler C., Kollmann K., Kantner I., Sexl V., Kleiter M. (2017). Estrogen Regulates Bone Turnover by Targeting RANKL Expression in Bone Lining Cells. Sci. Rep..

[74] Mishra S., Tai Q., Gu X., Schmitz J., Poullard A., Fajardo R.J., Mahalingam D., Chen X., Zhu X., Sun L.Z. (2015). Estrogen and estrogen receptor alpha promotes malignancy and osteoblastic tumorigenesis in prostate cancer. Oncotarget.

[75] Suva L.J., Washam C., Nicholas R.W., Griffin R.J. (2011). Bone metastasis: Mechanisms and therapeutic opportunities. Nat. Rev. Endocrinol..

[76] Smith M.R., Egerdie B., Hernandez Toriz N., Feldman R., Tammela T.L., Saad F., Heracek J., Szwedowski M., Ke C., Kupic A. (2009). Denosumab in men receiving androgen-deprivation therapy for prostate cancer. N. Engl. J. Med..

[77] Smith M.R., Eastham J., Gleason D.M., Shasha D., Tchekmedyian S., Zinner N. (2003). Randomized controlled trial of zoledronic acid to prevent bone loss in men receiving androgen deprivation therapy for nonmetastatic prostate cancer. J. Urol..

[78] Michaelson M.D., Kaufman D.S., Lee H., McGovern F.J., Kantoff P.W., Fallon M.A., Finkelstein J.S., Smith M.R. (2007). Randomized controlled trial of annual zoledronic acid to prevent gonadotropin-releasing hormone agonist-induced bone loss in men with prostate cancer. J. Clin. Oncol..

[79] Smith M.R., Saad F., Coleman R., Shore N., Fizazi K., Tombal B., Miller K., Sieber P., Karsh L., Damiao R. (2012). Denosumab and bone-metastasis-free survival in men with castration-resistant prostate cancer: Results of a phase 3, randomised, placebo-controlled trial. Lancet.

[80] Hussain M., Smith M., Sweeney C., Corn P., Elfiky A., Gordon M., Haas N., Harzstark A., Kurzrock R., Lara P. (2011). Cabozantinib (XL184) in metastatic castration-resistant prostate cancer (mCRPC): Results from a phase II randomized discontinuation trial. J. Clin. Oncol..

[81] Araujo J.C., Mathew P., Armstrong A.J., Braud E.L., Posadas E., Lonberg M., Gallick G.E., Trudel G.C., Paliwal P., Agrawal S. (2012). Dasatinib combined with docetaxel for castration-resistant prostate cancer: Results from a phase 1-2 study. Cancer.

[82] Bagnato A., Spinella F., Rosano L. (2008). The endothelin axis in cancer: The promise and the challenges of molecularly targeted therapy. Can. J. Physiol. Pharmacol..

[83] Qiao L., Liang Y., Li N., Hu X., Luo D., Gu J., Lu Y., Zheng Q. (2015). Endothelin-A receptor antagonists in prostate cancer treatment-a meta-analysis. Int. J. Clin. Exp. Med..

